# Approaches in rare diseases and pediatrics across international boundaries

**DOI:** 10.1186/1479-5876-10-S2-A43

**Published:** 2012-10-17

**Authors:** Michael Liebman

**Affiliations:** 1 Strategic Medicine, Inc., 231 Deepdale Dr Kennett Square, PA 19348, USA

## 

Rare diseases are designated as affecting less than 200,000 individuals (US) and of the approximately 7000 designated rare diseases, the majority of these occur in pediatric patients, and across international boundaries. An example is pediatric ARDS (Acute Respiratory Distress Syndrome) which is not diagnosed until a previously healthy child presents in the pICU with severe symptoms and in which more children die each year than from cystic fibrosis and leukemia, combined. The Nathaniel Adamczyk Foundation (NAF) is focused on identifying risk factors and opportunities for prevention of this devastating disease. Boththe diagnosis and patient management are challenged by having to deal with a syndrome in a critical care situation in a heterogeneous patient population.

NAF has undertaken the development of an (inter) national tissue and data repository to support both clinical research and enhanced clinical decision support for patient management. Creation of an analytical platform to integrate, access and analyze temporal clinical data ranging from the pICU to also incorporate neo-natal ICU and pregnancy history is underway with a prototype already in testing. Analytical methods are being evaluated in collaboration with Dr. Mike Quasney (Medical College of Wisconsin) and the Virtual Pediatric ICU and PALISI (Pediatric Acute Lung Injury and Sepsis Investigators). This effort is exploring expanded international partnerships in both Europe and China to increase the accessible data for analysis and to further participate in the development of better diagnostic standards.

We will present the initial state of both the analytics and platform development to encourage extension of this international effort to interested clinicians and clinical researchers. We believe that this unique approach, which focuses first on addressing the critical need to improve patient management through disease stratification, will not only benefit pARDS but be extensible to many other pediatric rare disorders.

